# Design and fabrication of multi-material broadband electromagnetic absorbers for use in cavity-backed antennas

**DOI:** 10.1016/j.heliyon.2023.e14164

**Published:** 2023-03-06

**Authors:** Ellen Gupta, Colin Bonner, Faheem Muhammed, Kyle McParland, Mark Mirotznik

**Affiliations:** a Electrical and Computer Engineering Department, University of Delaware, Newark, DE 19716, USA; b Department of Materials Science and Engineering, University of Delaware, Newark, DE 19716, USA

**Keywords:** Electromagnetic propagation in absorbing media, Graded RF absorption, Metamaterials, Multilayer structure, Additive manufacturing

## Abstract

We investigated the feasibility of designing and fabricating novel broadband radiofrequency (RF) absorbers for use in cavity-backed antennas. Fabricating the absorber involved a multi-material additive manufacturing (AM) approach that combined two polymer filaments: a low-loss dielectric filament and a lossy carbon-loaded filament. An iterative optimization algorithm was developed to deploy these filaments and create gradient distributions of material properties that minimize reflectance over a desired frequency band and a range of incident angles to achieve wideband electromagnetic absorption. The chosen material profiles were effectively realized using a spatially varying subwavelength lattice structure printed via fused filament fabrication. Experimentally, validation results demonstrated low reflectance over a wide frequency band, 10 to 40 GHz, and a range of incident angles, 0°–50°. Finally, this printed multi-material absorber was integrated within a cavity-backed spiral antenna and used to suppress backlobe radiation while maintaining an acceptable radiation pattern in the forward direction. While this study investigated cavity-backed antennas, these computational and experimental methods are potentially useful for a wide range of other applications.

## 1 Introduction

**Fig. 1:**
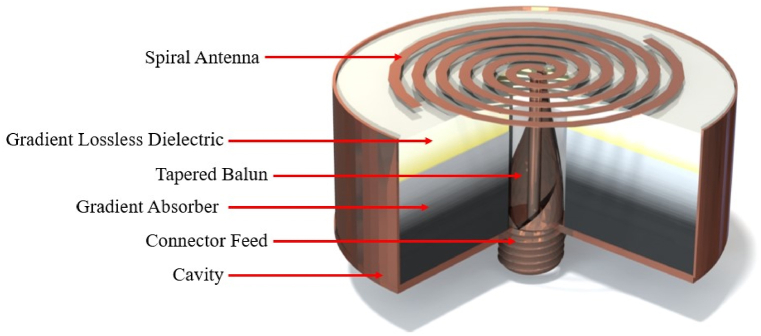
Illustration of the proposed additively manufactured absorber-loaded cavity-backed spiral antenna.

Electromagnetic (EM) absorbers are useful in a wide variety of EM devices and systems to minimize reflections from various dielectric and conductive surfaces. For antenna applications, absorbers play an essential role in reducing EM interference (i.e., EMI shielding) and preventing radiation in undesirable directions, such as being used for backlobe suppression. Examples of antennas often requiring backlobe suppression include planar spiral or sinuous antennas. These are wideband antennas without an integrated ground plane that consequently generate high backlobe radiation, which can potentially cause undesirable interference with itself or other antennas on the same platform. A common approach for resolving this issue is surrounding the entire backside of the antenna with a metallic cavity filled with EM absorbing material (see Fig. 1). This absorber minimizes reflections from the cavity's metallic surfaces, otherwise causing detrimental frequency-dependent interference. One of the design and manufacturing challenges of creating this cavity is optimizing the absorber's performance within the size and frequency constraints. This work will show how multi-material additive manufacturing (AM) can serve as an effective tool to create high performing wideband absorbers that can be tailored both electromagnetically and geometrically for a wide range of applications, including cavity-backed antennas. In addition to achieving good wideband performance, using multi-material AM provides additional advantages, such as creating geometrically complex shapes with good repeatability and low cost.

EM absorbers are generally classified as either resonant or broadband. Resonant absorbers combine one or more thin layers of lossy resonant structures, such as frequency selective surfaces, typically spaced with a quarter wavelength between layers. Examples of resonant absorbers include Salisbury screens, Jaumann absorbers, and circuit analog absorbers [1,2]. Broadband absorbers achieve ultra-wideband performance by using a material in which the complex impedance is continuously varied or graded according to a predetermined profile. Various profiles are commonly used, including linear, exponential, and Klopfenstein. These gradient dielectric absorbers can be designed with low reflectance over a wide frequency band approaching the theoretical limits [[3], [4], [5], [6], [7], [8]]. To achieve a continuous EM impedance profile $\eta^{*}\left(x\right)$, the material properties of the absorber, relative complex permittivity $\varepsilon_{r}^{*}$ and/or permeability $\mu_{r}^{*}$, are varied in the through-thickness direction, x, according to

(1) $$\eta^{*}\left(x\right)=\eta_{o}\sqrt{\frac{\mu_{r}^{*}\left(x\right)}{\varepsilon_{r}^{*}\left(x\right)}}$$

where $\eta_{o}$ denotes the intrinsic impedance of free space. It should be noted that for non-magnetic absorbers, $\mu_{r}^{*}=1.0$. The current difficulty in addressing these spatially graded EM properties lies in the materials and fabrication methods employed.

One common approach to constructing graded absorbers uses a discrete number of layers where the EM properties of each layer are optimized. However, this approach is practical only for a few layers since it depends on the ability to synthesize multiple materials that each have the required EM properties. A second approach uses a geometrical transition formed within a homogenous lossy material, such as carbon-loaded foam to produce an effective graded impedance, often seen as a pyramidal structure. An example is the pyramidal absorbers commonly used in anechoic chambers. While quite effective, this absorber type is large and difficult to use on complex non-planar geometries. A third approach is to coat the surface of a porous material, such as a honeycomb, with a lossy surface coating that varies in thickness as a function of distance from the surface. This is generally achieved by successively dipping the honeycomb in a coating material to various depths. In addition to being a slow, tedious process, this method can produce considerable sample-to-sample variability. Thus, a more robust and reliable method for fabricating graded dielectric absorbers is needed.

Multi-material AM offers an innovative approach for fabricating graded absorbers that combines many of the advantages of the previously discussed methods with the potential for improved reliability and lower cost. While the relevant literature is admittedly sparse, a few investigations have explored the use of AM for the fabrication of graded EM absorbers. Researchers have printed a honeycomb structure to develop a broadband EM wave absorber that can be difficult to manufacture using traditional methods [9]. The authors in Ref. [10] used AM to print pyramidal absorbing structures using carbon-loaded polymer filaments. They demonstrated the ability to fabricate complex pyramidal patterns of space-filling curves that effectively absorbed incident radiation in the high millimeter wave band (63 to 215 GHz). The authors in Ref. [11] described an AM approach to realize multilayer graded absorbers by varying either the fill fraction or the local fill fraction of a reduced graphene oxide loaded polylactic acid (PLA). A carbon or graphene-loaded filament is often chosen due to its associated high loss tangent. These loaded materials continue to be explored for their absorbing qualities and how they are manufactured using AM [[12], [13], [14], [15], [16]].

**Fig. 2:**
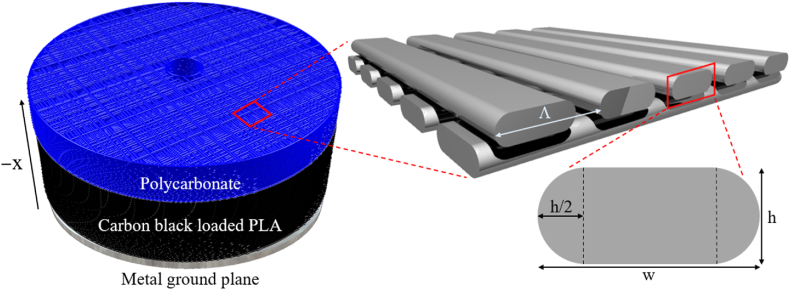
Multi-material AM approach proposed for wideband graded absorber in cavity-backed antennas. The concept combines a low-loss polycarbonate antireflecting layer and lossy carbon-loaded PLA for EM attenuation. Graded effective properties are achieved using spatially varying subwavelength lattice of FFF printed in a log-pile structure by changing Λ in the x direction.

This research uses a multi-material AM approach that builds on these previous studies by combining a low-loss polymer filament with a much higher loss carbon-loaded filament to manufacture multi-material graded EM absorbers (see Fig. 1, Fig. 2). The low-loss material was used as a wideband antireflective layer that transitioned into the higher loss material for energy absorption. This approach is shown to be capable of wideband performance between 4 and 40 GHz and can be geometrically tailored for use in absorber-loaded cavity-backed antennas. To develop the absorber, a thorough material characterization study of commercially available polymer filaments was conducted. An iterative optimization algorithm was then developed to design the specific complex permittivity, $\varepsilon_{r}^{*}\text{,}$ profiles for both the low and high-loss materials. To realize the desired permittivity distributions fused filament fabrication (FFF) was used to create a spatially varying subwavelength lattice where the effective $\varepsilon_{r}^{*}$ was varied to match desired properties. To demonstrate the functionality of this AM design process for antennas, a cavity-backed spiral antenna was designed and additively manufactured. While this absorber was designed for a cavity system, experimental measurements show promising results that can be extended to many more EM applications using the same optimization design algorithm.

The outline for the subsequent portions of the paper is as follows. Section 2 describes the experimental methods used to select and evaluate the filament materials and their measured radiofrequency (RF) properties. This section also describes the specific algorithms used to design the detailed absorber structure given the selected materials and specific print parameters. Experimental results are then presented that validate the design approach. Section 3 describes how the absorber was integrated within a cavity-backed spiral antenna. This includes how the absorber was modified and designed to fit on the back of the spiral antenna and how this antenna system was measured and compared to predictions. Finally, Section 4 concludes this paper and discusses further work that can be implemented.

## 2 Design, fabrication, and validation of graded dielectric absorber

This section describes the overall concept, materials, and methods used to realize the broadband graded dielectric absorbers and numerical and experimental validation results.

### 2.1 Concept and materials

The basic design concept is illustrated in Fig. 2. Here, FFF is used to create a spatially varying lattice structure that geometrically changes the average material density. If this variation is on a scale much smaller than the wavelength (e.g., Λ≪λ, where Λ is the unit cell periodicity shown in Fig. 2), then the local $\varepsilon_{r}^{*}$, from Eq. (1), can be approximated by an effective permittivity, $\varepsilon_{eff}^{*}$, that is dependent on the local volume fraction (VF) of the material and the specific lattice geometry. In this study, the lattice geometry is the simple log-pile structure illustrated in Fig. 2. Specifically, each printed layer is composed of parallel filaments separated by a fixed distance. Filaments in adjacent layers are printed orthogonally to minimize polarization dependence in the transverse plane. Since the layer height, *h*, is much smaller than the wavelength, the effective material properties of the printed structure can be considered nearly continuous in the through-thickness direction. The cross-sectional geometry of the printed filaments shown in Fig. 2 models the shape extruded from a circular FFF nozzle. For this geometry, the cross-sectional area of the printed filament can be calculated using

(2) $$A_{cross}=\left(w-h\right)∙h+\pi {\left(\frac{h}{2}\right)}^{2}$$

where *w* and *h* are the width and height, respectively, of the print path as represented in Fig. 2. VF of a printed layer is then calculated using

(3) $$VF=\frac{A_{cross}}{h\Lambda }=\frac{w-\left(1-\frac{\pi }{4}\right)h}{\Lambda }$$

where Λ is also known as pitch, or repeating distance, of the printed filaments. Since the filament's $A_{cross}$ and *h* are fixed, the volume fraction is set by varying Λ from layer-to-layer.

The effective $\varepsilon_{r}^{*}$ not only depends on the local VF and lattice geometry but also on the filament's intrinsic material properties. Namely, the $\varepsilon_{r}^{*}$ is given by

(4) $$\varepsilon_{r}^{*}=\varepsilon^{\prime }-j\;\varepsilon^{\prime \prime }\;{=\varepsilon }_{r}∙\left(1-j\;\tan\;\delta \right)$$

where $\varepsilon_{r}$ denotes the material's dielectric constant and tanδ indicates its loss tangent, and where $\varepsilon^{\prime }$ and $\varepsilon^{\prime \prime }$ are the real and imaginary permittivity values. For this study, two different commercially available polymer filaments were utilized. The first was Polymaker's PolyLite™ polycarbonate (PC). This material was measured previously and found to be non-dispersive up to 40 GHz with a dielectric constant of $\varepsilon_{r}$ = 2.68 and a low-loss tangent of tanδ = 0.002 [17]. PC was chosen to function as the antireflection or impedance matching layer at the front surface of the absorber [18]. The second material was a lossy conductive filament, Protopasta's Composite Conductive Fiber PLA CDP11705, which is a 20% carbon black (CB) loaded PLA. Due to the presence of the CB, the PLA filament has a high enough loss tangent capable of attenuating RF waves. Notably, multiple carbon-loaded filaments were characterized and assessed in this study, however, Protopasta's CDP11705 was found to result in RF properties within a range well suited for wideband graded absorber designs. The following sections describe the specific experimental methods used to characterize the $\varepsilon_{r}^{*}$ of the CB-loaded PLA material.

### 2.2 Fabrication

The calibration samples, prototype absorbers, and cavity-backed antennas were fabricated using a nScrypt 3Dx-700 multi-material printer with a FFF print head. This manufacturing method provides an advantage due to the layer-by-layer print approach that tailors the absorber in thin discrete layers. A nozzle diameter of 0.3 mm was employed, and the filament was printed with a layer height of 0.15 mm for each material at 15 mm/s. Protopasta's conductive PLA was printed at 225 °C with the print bed temperature at 60 °C, while Polymaker's PC was printed at 300 °C with the print bed temperature of 100 °C. Each material was printed separately and combined after printing. After initial prototypes were fabricated, the RF material properties of each material were characterized following the steps described in Section 2.3. To print the spatially variable infill patterns, a custom slicer program was developed using MATLAB®. This program generates GCODE directly from an STL file and a desired graded VF distribution. This allowed a unique infill pattern to be applied and printed in nearly any arbitrary shape. However, before the graded absorbers could be designed and printed, samples of constant infilled plates were printed and characterized to determine the intrinsic EM properties of the CB-loaded polymer filaments.

### 2.3 Material characterization of lossy CB loaded polymer filaments

**Fig. 3:**
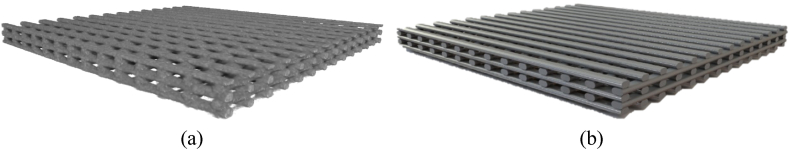
(a) A CT scan of the log-pile AM part and (b) a schematic of the desired log-pile AM part.

This section describes the experimental and analytical approach used to extract the CB loaded PLA's frequency-dependent EM properties. Five different calibration samples were fabricated to characterize the material experimentally. The log-pile lattice structure within each sample was fixed, resulting in an effectively homogenous plate. However, sample-to-sample VF was varied from a minimum value of 0.09 to a high value of 0.85 with the specific print parameters provided in Table 1. To generate a particular VF, Λ was varied while *w* and *h* were fixed at 0.3 mm and 0.15 mm, respectively. Each calibration plate was 203.2 mm × 203.2 mm x 0.9 mm in size, corresponding to six printed layers. To ensure that the printed geometries were consistent with the geometrical model shown in Fig. 2 and described by Eqs. (2), (3), detailed imaging using micro-computerized tomography (CT) was conducted (see Fig. 3) for each calibration sample. For this, a Rigaku® GX 130 CT Scanner was used with a scan time of 14 min over a field of view of 10 mm. Edge lengths, statistical void properties, and volumetric imaging were processed through Dragonfly 2021.3 by Object Research Systems. To determine the porosity of the samples, Otsu's method was utilized in which pixels were separated into two classes, void or composite, by minimizing intra-class variance [19]. Measurements of *w* and *h* were also taken and found to be consistent throughout each calibration sample. The image processed VF, and the calculated VF using Eq. (3) of each printed sample are presented in Table 1. The processed VF was found to match the calculated VF closely. The VF calculated from Eq. (3) will be used to characterize the EM properties. Fig. 3 shows the accuracy of using the measured CT results, Fig. 3a, to model the geometry of the AM lattice structure, Fig. 3b.

**Table 1:** Realized VF of 3D printed calibration samples.

CT Scan Data	Measured VF	Calculated VF using (3)	Pitch Λ (mm)
	0.091	0.089	3.00
	0.179	0.179	1.50
	0.359	0.358	0.75
	0.539	0.537	0.50
	0.850	0.850	0.315

#### 2.3.1 Material RF characterization

**Fig. 4:**
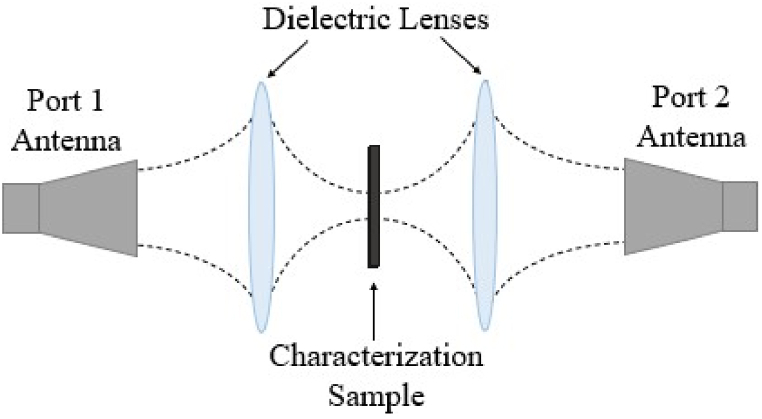
Free-space focused beam materials measurement system capable of testing transmission through material sheets between 4 GHz and 40 GHz.

To measure the $\varepsilon_{r}^{*}$ of the calibration samples described in Table 1, a custom-built free-space focused beam measurement system was used (see Fig. 4) [20]. This system consisted of two 24 inch diameter lenses focusing the beam between two linearly polarized horn antennas, with the polarization aligned with the principal axes of the lattice structure. The samples where The following algorithm was applied to extract the material properties of the CB-loaded filament from the measured data. First, the $\varepsilon_{r}^{*}$ of the lossy filaments, $\varepsilon_{cf}^{*}$, was assumed to follow a simple dispersion relationship given as

(5) $$\varepsilon_{cf}^{*}=\varepsilon_{\infty }+\frac{B}{1+j2\pi f\tau }-j\frac{\sigma }{2\pi f\varepsilon_{o}}$$

where, $\varepsilon_{\infty }$, *B*, τ, and σ are unknown constants to be determined, and *f* is frequency. The next step was to model $\varepsilon_{eff}^{*}$ of the calibration samples when the CB filaments were printed in a log-pile configuration. For that model, the well-known Maxwell-Garnett mixture formulas were employed [21] to arrive at

(6) $$\varepsilon_{eff}^{*}=\frac{1}{2}\left[\left(VF∙\varepsilon_{cf}^{*}+\left(1-VF\right)\right)+\frac{\varepsilon_{cf}^{*}\left(1+VF\right)+\left(1-VF\right)}{\varepsilon_{cf}^{*}\;\left(1-VF\right)+\left(1+VF\right)}\right]$$

**Fig. 5:**
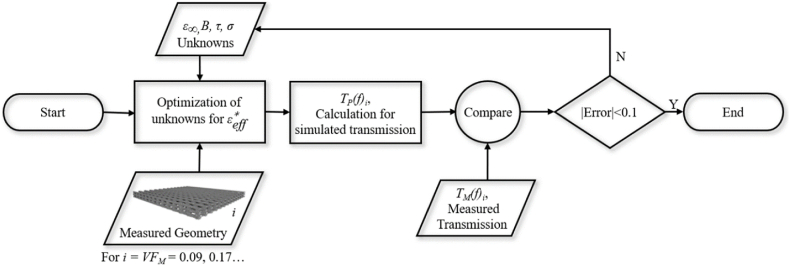
Flowchart diagrams the computational methods deployed to optimize $\varepsilon_{eff}^{*}$ for the CB-loaded PLA.

**Fig. 6:**
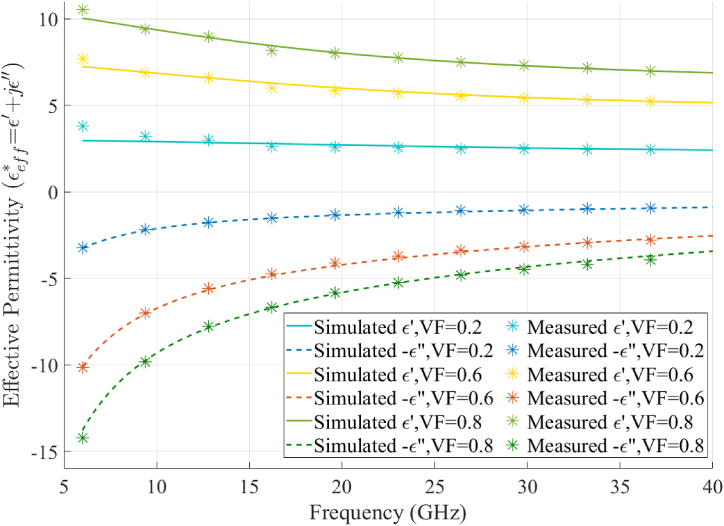
The simulated and measured $\varepsilon_{eff}^{*}$ for the CB-loaded filament at multiple VF. The real ($\varepsilon^{\prime }$) and imaginary ($\varepsilon^{\prime \prime }$) values for dispersive permittivity are plotted.

It should be noted that Eq. (6) averages the effective $\varepsilon_{r}^{*}$ of filaments both parallel and perpendicular to the direction of polarization to model the specific geometry of the log-pile lattice. Substituting Eq. (5) into Eq. (6) derived an effective media model for the printed calibration samples described in Table 1. The final step was to estimate the four unknown constants in Eq. (5) by fitting the effective media model to the measured permittivity data using the optimization algorithm described in Fig. 5. This algorithm found the set of values given in Table 2 that fully model the frequency dependent $\varepsilon_{r}^{*}$ of the CB-loaded filaments. Fig. 6 compares the measured and simulated $\varepsilon_{r}^{*}$ for samples printed at several different VFs. Two important observations should be highlighted based on the data shown in Fig. 6. First, the effective media model described in Eq. (5), Eq. (6), and Table 2 can be used to accurately predict the $\varepsilon_{eff}^{*}$ over a wide range of VFs and operating frequencies. Second, $\varepsilon_{r}$ and tanδ, from Eq. (4), can be predictably varied over a wide range of values by simply varying the geometry of the printed lattice structure.

**Table 2:** Calculated dispersion coefficients, for Eq. (5), for CB-loaded PLA (CDP11705).

Unknown	$\varepsilon_{\infty }$	B	τ (nsec)	σ (S/m)
Value	11.2	9.8	0.0758	11.5

### 2.4 Absorber design

**Fig. 7:**
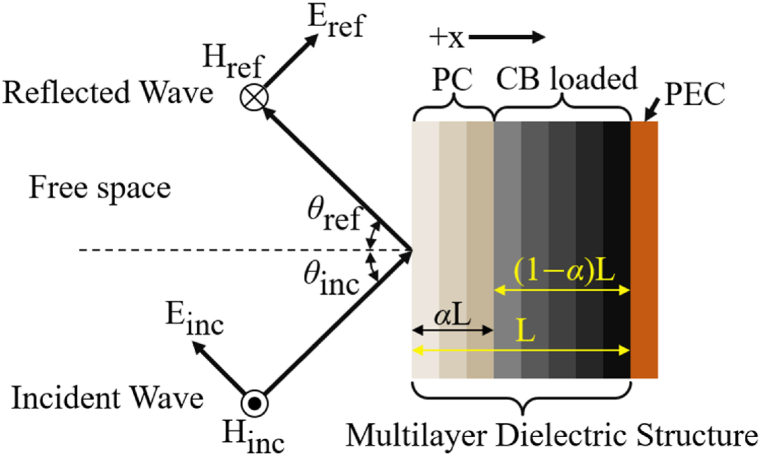
Multi-slab approach for impedance matching EM wave absorber.

After the filament's RF material properties had been thoroughly characterized, an optimization algorithm was developed to design graded multi-material absorbers, using the same print parameters as the calibration samples from the previous sections. The absorber was assumed to be a planar multilayered stack of total thickness L, as illustrated in Fig. 7, backed by a perfect electrically conducting (PEC) ground plane. Specifically, the structure was composed of a front PC layer of thickness α L and a CB-loaded layer of thickness (1 −α)L. Here the thickness parameter α was considered a free parameter, 0 < α < 1, determined during the optimization process. All calculations assumed a monochromatic plane wave illumination at an incident angle of $\theta_{inc}$, and transverse magnetic (TM) polarization.

**Fig. 8:**
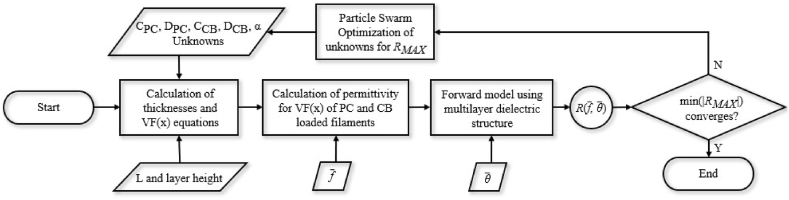
Flowchart diagrams the computational methods deployed to optimize the PC and CB- loaded layer gradients to minimize reflection.

A flow diagram illustrating the optimization algorithm is shown in Fig. 8. The objective was to determine the spatially varying volume fraction VF(x) for both the PC and CB-loaded materials, and the thickness parameter α that will minimize reflectance, R_*MAX*_, given a desired frequency band, range of incident angles, and total absorber thickness.

**Fig. 9:**
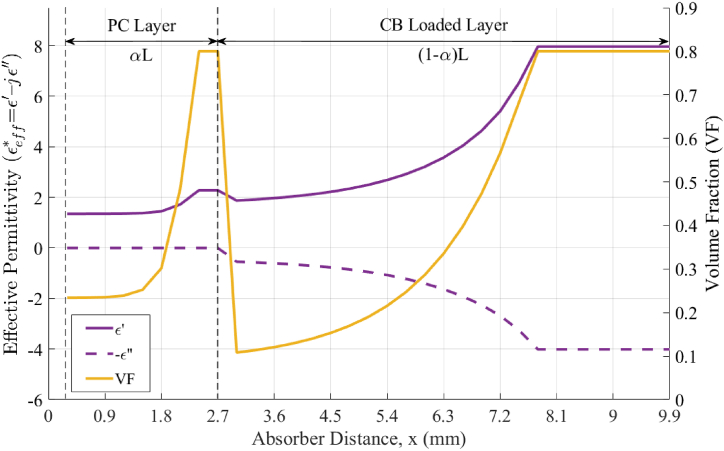
VF and effective $\varepsilon_{eff}^{*}$ for the PC layer and CB loaded layer as a function of the through-thickness distance x at 15 GHz.

To better constrain the problem, VF(x) for both the PC and CB-loaded materials were assumed to follow an exponential taper function given by

(7) $${VF\left(x\right)}_{ii}=\min\left({VF}_{\min}+C_{ii}{(e}^{D_{ii}\left(x/L\right)}-1),{VF}_{\max}\right),ii=PC\;or\;CB$$

where C_*ii*_ and D_*ii*_ are unknown parameters to be optimized. In Eq. (7), ${VF}_{\min}$ and ${VF}_{\max}$ represent the minimum and maximum achievable infill values, respectively, determined by the specific printer and print parameters used. This ensures that the resultant design is manufacturable. Fig. 9 illustrates a typical VF distribution and its corresponding $\varepsilon_{eff}^{*}$ as calculated by Eqs. (5), (6) using incident angles ranging from 0 to 60° and with the permittivity values calculated at 15 GHz.

Initial values were assigned to the five unknown parameters (i.e., C_PC_, D_PC_, C_CB_, D_CB_ and α) to start the iterative optimization algorithm. Using Eq. (7), VF values were sampled at every other print layer thickness. This sampling method was necessary to represent the two layers of the log-pile structure illustrated in Fig. 2. For each VF distribution, the $\varepsilon_{eff}^{*}$ gradient was found using Eqs. (5), (6). A multilayered dielectric solver was then used to calculate the reflectance R for a range of input frequencies and incident angles supplied by the user seen in Fig. 8 [22]. Finally, a particle swarm optimization algorithm was employed to determine the five unknown parameters to minimize the maximum reflectance over the desired range of frequencies and incident angles, as seen in Fig. 8.

### 2.5 Illustrative examples

**Fig. 10:**
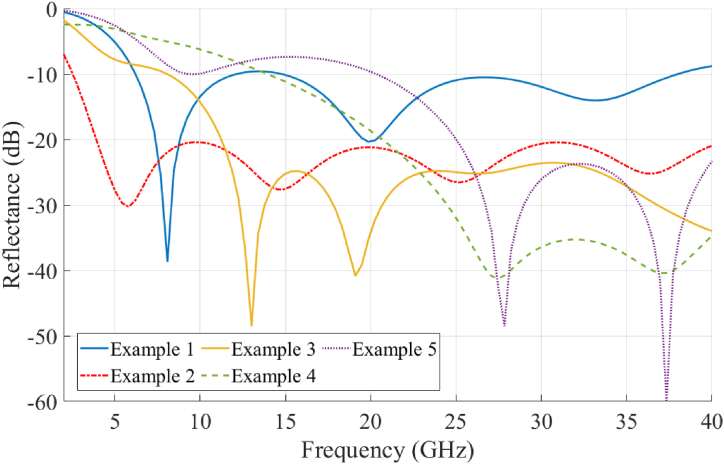
Simulated reflectance of graded multi-material absorbers described in Examples #1 through #5 in Table 3 at normal incidence.

The illustrative examples described in Table 3 were designed and numerically simulated to evaluate the performance and flexibility of this multi-material AM approach. The measured properties of the PC and CB-loaded materials were used, and fabrication constraints, in terms of the minimum and maximum allowable VF, were enforced to ensure that each design was manufacturable. These specific examples were chosen to test the algorithm's ability to create graded absorbers that operate over a wide range of frequencies. The calculated reflectance at normal incidence for each of the examples is plotted in Fig. 10. As with any EM absorber, there are fundamental limits to the minimum reflectance that can be achieved over an operating bandwidth given a specific absorber thickness. In Example #1, the goal was to obtain a reflectance below −20 dB over the wide operating frequency range of 4 to 40 GHz. To accomplish this, a multi-material absorber 36 mm in thickness corresponding to an electrical thickness of 0.48*λ*_max_ was needed. Here, *λ*_max_ refers to the free-space wavelength at the lowest operational frequency. However, for a narrow band design such as Example #2, a reflectance below −20dB can be achieved with an absorber of only 0.15*λ*_max_. The Ku-band design of Example #3 was entirely manufactured, tested, and integrated as part of the cavity-backed spiral antenna. As a result, the frequency range of 12 to 18 GHz was chosen to match the operational band of the spiral antenna. The total thickness of ∼10 mm was selected to fit within the metallic cavity illustrated in Fig. 1. The VF and $\varepsilon_{eff}^{*}$ distributions for this design is shown in Fig. 9.

**Table 3:** Simulated absorber design examples at multiple frequency bands.

	Example #1	Example #2	Example #3^a^	Example #4	Example #5
Frequency range, GHz	7–8	4–40	12–18	26–40	26–40
Total thickness, L (mm)	6.6 (0.15λ_max_)	36 (0.48λ_max_)	9.9 (0.4λ_max_)	9.9 (0.86λ_max_)	4.5 (0.39λ_max_)
α	0.286	0.308	0.276	0.092	0.282
C_PC_/D_PC_	89.37/88.36	0.13/1.87	6.1e-5/11.98	66.72/0.0001	38.42/5.09
C_CB_/D_CB_	2.77/0.0001	0.0005/10.51	0.02/5.25	0.03/11.37	0.84/0.91

a Indicates the design parameters of the absorber used for experimental validation.

### 2.6 Experimental validation of graded dielectric absorbers

**Fig. 11:**
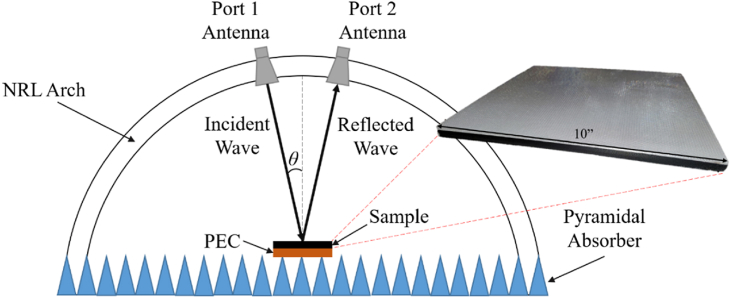
Free space NRL arch system used to measure reflectance as a function of frequency and incident angle.

To experimentally validate the design approach, a test plate corresponding to the structure described in Example #3 of Table 3 was fabricated and then measured using a free-space Naval Research Laboratory (NRL) arch system, as illustrated in Fig. 11 [23]. The plate was 254 mm × 254 mm x 9.9 mm in size, corresponding to ten free-space wavelengths on a side at the lowest frequency of interest. The sample was placed in the center of the arch on a metal plate of the exact dimensions to simulate the absorber over a conductive ground plane. The NRL arch was then used to measure the reflectance over a wide frequency band, 4 to 40 GHz, and over incident angles varying from 10^o^ to 50^o^. For these measurements, the polarization of the incident field was set to linear horizontal or TE polarization. Two configurations of the absorber were evaluated. One used a complete multi-material approach that combined the PC and CB-loaded PLA layers. The other used the more traditional approach of a single lossy material absorber consisting of only the CB- loaded PLA layer. Pyramidal absorbers were placed around the sample to minimize edge effects and ground reflections.

**Fig. 12:**
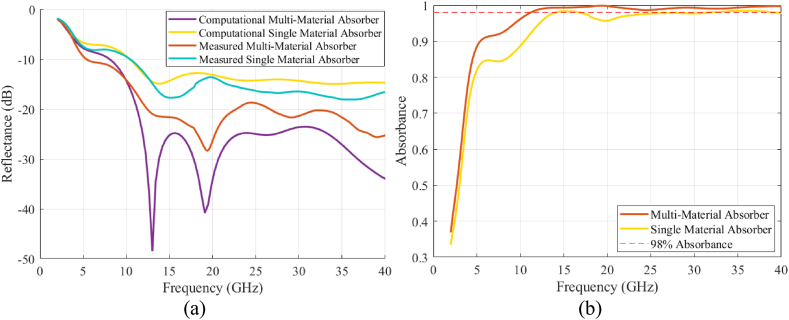
(a) Comparison of measured reflectance (using the NRL arch system shown in Fig. 11) and simulated reflectance with an incident angle of 10^o^ off normal. Results are provided for a multi-material (PC and CB) absorber and a single material graded (CB) absorber. (b) Measured absorbance for both absorbers on a linear scale illustrating >98% energy absorbance over the desired frequency band for the multi-material absorber.

Fig. 12a compares the measured reflectance data at an incident angle of 10^o^ to the simulated results as a function of frequency. Results are shown for both the full multi-material absorber with PC and CB layers, and the CB-loaded PLA sample alone. Both cases demonstrated reasonable agreement between measured and simulated data. The measurement results also showed extremely promising results from having a reflectance of under −20 dB over the frequency bands of interest. The conductor-backed multi-material absorber achieved a reflectance below −10 dB over the entire 6 to 40 GHz frequency range and below −15 dB from 10 to 40 GHz. The difference between the measured and simulated results, particularly the deep nulls seen within the simulated results, was most likely due to the finite size of the sample plate. In contrast, the measured results from the single material CB-loaded PLA could not achieve a reflectance below −20 dB at any frequency and were generally more than 5 dB higher than the multi-material design. This demonstrates the advantage of integrating the low-loss antireflective layer to realize wideband absorption. In Fig. 12b, the absorbance is provided on a linear scale to highlight that over 98% of the incident energy is absorbed for the multi-material design over the frequency range of ∼10 to 40 GHz.

**Fig. 13:**
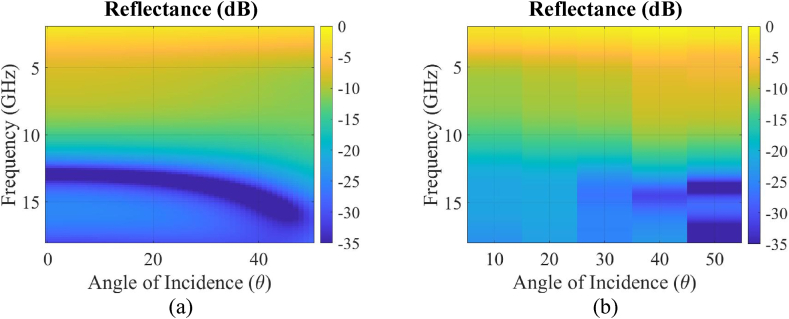
(a) Simulated and (b) measured reflectance as a function of incident angle and frequency between 4 GHz and 18 GHz.

The simulated and measured reflectance as a function of frequency and incident angle for the multi-material absorber is shown in Fig. 13a and b, respectively. The results demonstrated low reflectance (<-15 dB) and a high rate of absorption up to 50^o^ off-normal within the operational frequency band of 12 to 18 GHz. While there is a reasonably good match between measured and simulated results, some deviation was observed particularly at larger incident angles. These errors are likely due to the horn antenna's illumination region growing in size as the incident angle increases. This will result in enhanced edge scattering from the ground plane. Despite these errors, the trend remained the same and within the desired frequency band.

## 3 Design, fabrication, and characterization of cavity-backed spiral antenna

### 3.1 Antenna design and fabrication

The previous sections described the graded multi-material absorber design methodology and experimental validation. This section describes how the absorber was integrated within the spiral antenna assembly illustrated in Fig. 1.

**Fig. 14:**
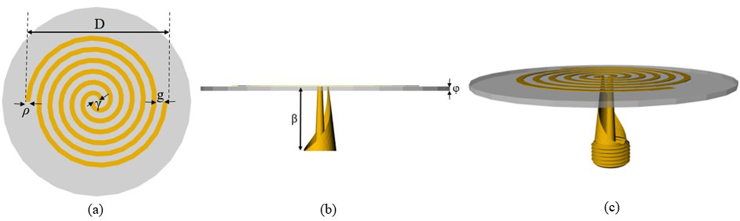
(a) Top, (b) side, and (c) isometric views of the spiral antenna design.

The spiral antenna design is based on the work in Refs. [24,25]. This fully 3D printed integrated antenna feed combines: a threaded SMA connector, a tapered coaxial balun, and a 4-turn Archimedean spiral antenna. The balun, described in Refs. [24,25], transforms the 50 Ω connector impedance to the 160 Ω input impedance of the spiral over the Ku-band. The dimensions and parameters of the spiral antenna and coaxial feed are given in Table 4 and Fig. 14a–c. The integrated antenna feed was fabricated using the nScrypt™ multi-material AM system. All dielectric regions were fabricated using polycarbonate via FFF, and all conductive regions were printed via micro-dispensing silver-based inks.

**Table 4:** Spiral antenna parameters.

Arm width (ρ)	1.25 mm
Gap width (g)	0.9 mm
Spiral diameter (D)	29 mm
Feed gap (γ)	0.95 mm
Balun length (β)	7.5 mm
Substrate thickness (φ)	1.6 mm
Substrate permittivity	1.75

**Fig. 15:**
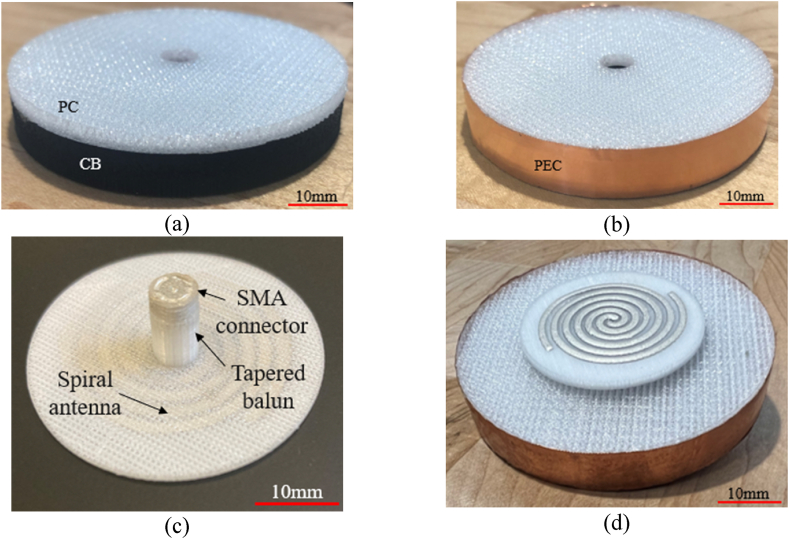
(a) AM fabricated annular multi-material absorber, (b) multi-material absorber integrated within a metallic cavity, (c) printed spiral antenna with integrated tapered balun and threaded SMA connector, and (d) fully integrated cavity-backed spiral antenna with the printed absorber.

Using this spiral antenna design, the absorber described in Example #3 from Section 2.5 was modeled and printed as the annular structure seen in Fig. 15a. The inner diameter was sized to fit the feed network of the antenna. The outer diameter and the air gap between the back of the spiral antenna substrate and the top face of the absorber were determined numerically using the commercial EM solver COMSOL Multiphysics®. This EM solver optimized the cavity dimensions to minimize the backlobe radiated by the cavity-backed spiral antenna and calculated a cavity of 60 mm in diameter and 9.9 mm in thickness, as shown in Fig. 15a and b. Lastly, the integrated antenna feed, as seen in Fig. 15c, was manually inserted within the inner opening of the absorber/cavity assembly producing the final absorber-loaded cavity-backed antenna prototype shown in Fig. 15d.

### 3.2 Experimental characterization

This section describes the experimental characterization results from the cavity-backed antenna shown in Fig. 15. The measurements include evaluations of return loss, normalized radiation patterns, and axial ratio.

#### 3.2.1 Return loss measurements

**Fig. 16:**
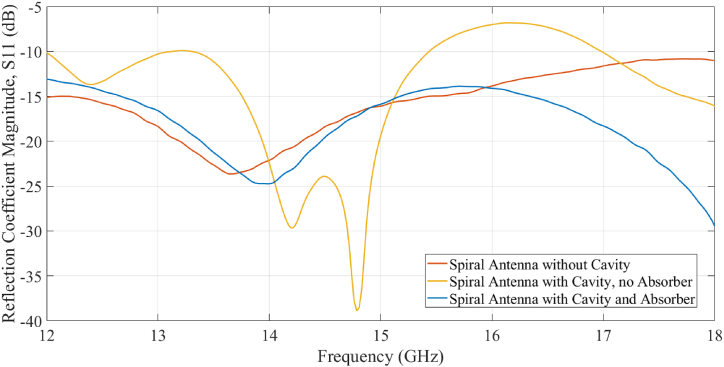
Reflection coefficient magnitude S11 of multiple antenna configurations: the spiral antenna alone without a cavity, the spiral antenna backed with a metal cavity and no absorber, and the spiral antenna backed with the absorber and metal cavity.

Return loss was measured using an Agilent E8361C vector network analyzer. Fig. 16 compares the return loss for multiple system configurations to further explore how the spiral antenna works in parallel with the RF absorber. The configurations include the spiral antenna alone, the spiral antenna backed with a metal cavity but no absorber, and the spiral antenna backed with an absorber-loaded cavity. The magnitude of the reflection coefficient (S11) is shown in Fig. 16 between 12 and 18 GHz.

The spiral antenna with no cavity demonstrated a low return loss (<10 dB) over a reasonably wide frequency band (12 to 18 GHz). However, when the antenna was placed in the metallic cavity without the printed absorber, frequency dependent oscillations in return loss appeared. This is almost certainly due to reflections from the bottom and sides of the cavity resulting in higher return losses and thus lower radiation efficiency within the frequency band of interest. When the graded absorber was included in the antenna assembly, these frequency dependent oscillations were not observed, indicating that the absorber successfully attenuated incident radiation and prevented reflections from the cavity surfaces. Surprisingly, the absorber-loaded cavity configuration resulted in a lower return loss than the spiral antenna alone above 16 GHz. This unexpected result is believed to be due to the absorber preventing higher order radiation modes generated in the balun and connector from interfering with the spiral antenna. However, further studies are currently underway to fully understand this phenomenon.

#### 3.2.2 Measured radiation patterns and axial ratio

**Fig. 17:**
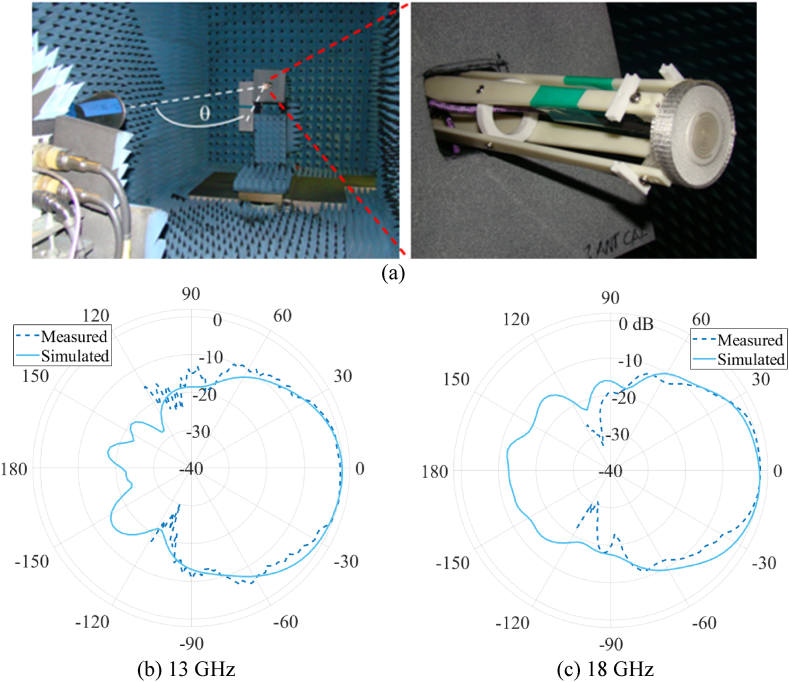
(a) Anechoic chamber at Cobham Advanced Electronic Solutions (CAES), https://caes.com/, used to conduct radiation pattern measurements. Measured and simulated results for (b) vertically polarized normalized gain at 13 GHz, inside the target frequency band and (c) vertically normalized gain at 18 GHz at the maximum value used for the target frequency band.

**Fig. 18:**
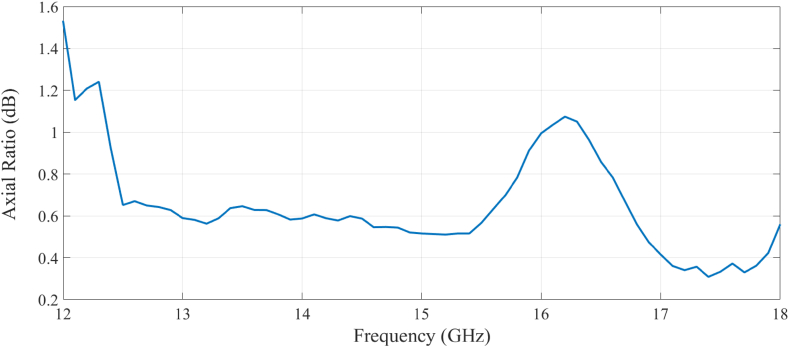
Measured axial ratio of the multi-material cavity-backed spiral antenna at boresight between 12 and 18 GHz.

Radiation pattern measurements were conducted at CAES in Lansdale, PA, in an anechoic chamber at frequencies spanning 12 to 18 GHz and at elevation angles, θ, varying from −120° to 120° from boresight, as seen in Fig. 17a. Results for both vertical and horizontal linear polarizations were measured directly. Results for circular polarization, such as axial ratio, were calculated during post-processing. These data measurements are presented in Fig. 17b and c and compared against simulated data for normalized gain patterns from the absorber-loaded cavity-backed spiral antenna at 13 GHz and 18 GHz. The results show a close match between measured and simulated radiation patterns. The simulated results demonstrate that the backlobe radiation is successfully suppressed with normalized gain values remaining −10 dB (i.e., >90% absorption) below the boresight gain. The forward radiation pattern maintained its gain, and the antenna's main lobe was not distorted. This spiral antenna system with the absorber also maintained an acceptable axial ratio below 3 dB, as shown in Fig. 18.

## 4 Conclusions

This work fully details the design, fabrication methods, and materials used to realize wideband EM absorbers for use in cavity-backed antennas. The multi-material absorber design combines a low-loss polycarbonate filament with a lossy carbon-loaded PLA filament to achieve wideband impedance matching and absorption. The low-loss material was useful as a transition layer that greatly improves impedance matching allowing for wideband absorbance. An iterative optimization algorithm was developed to calculate the specific graded material profiles needed to minimize reflectance over a desired frequency band and range of incident angles. A spatially varying subwavelength lattice structure was printed via FFF with varying fill fractions to effectively realize the graded material properties. The design was evaluated experimentally and demonstrated low reflectance over a wide frequency band (∼10 to 40 GHz) and a range of incident angles (0^o^ to 50^o^). Finally, this printed multi-material absorber was integrated within a cavity-backed spiral antenna and used to effectively suppress backlobe radiation while maintaining an acceptable radiation pattern in the forward direction. Thus, the fabrication of a multi-material broadband RF absorber is feasible using AM and the approach demonstrated here. This computational and experimental method can be applied to various loaded polymer filaments assuming the material is appropriately and fully characterized. Moreover, it can be used to fabricate absorbers with various desired material profiles and can be expanded for use in various other applications, such as antenna isolation and mitigation.

## Author contribution statement

Ellen Gupta, Mark Mirotznik: Conceived and designed the experiments; Performed the experiments; Analyzed and interpreted the data; Contributed reagents, materials, analysis tools or data; Wrote the paper.

Colin Bonner, Faheem Muhammed, Kyle McParland: Performed the experiments; Analyzed and interpreted the data; Contributed reagents, materials, analysis tools or data.

## Funding statement

This research did not receive any specific grant from funding agencies in the public, commercial, or not-for-profit sectors.

## Data availability statement

Data included in article/supplementary material/referenced in article.

## Declaration of interest’s statement

The authors declare no conflict of interest.
